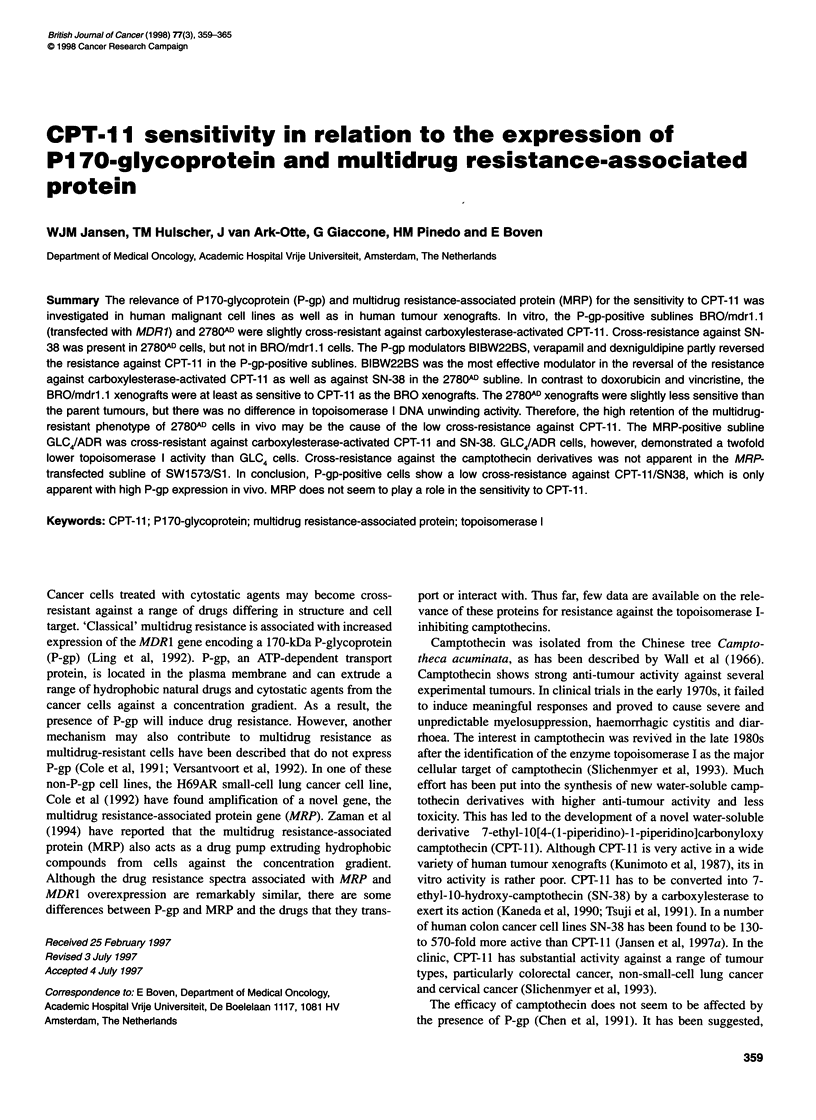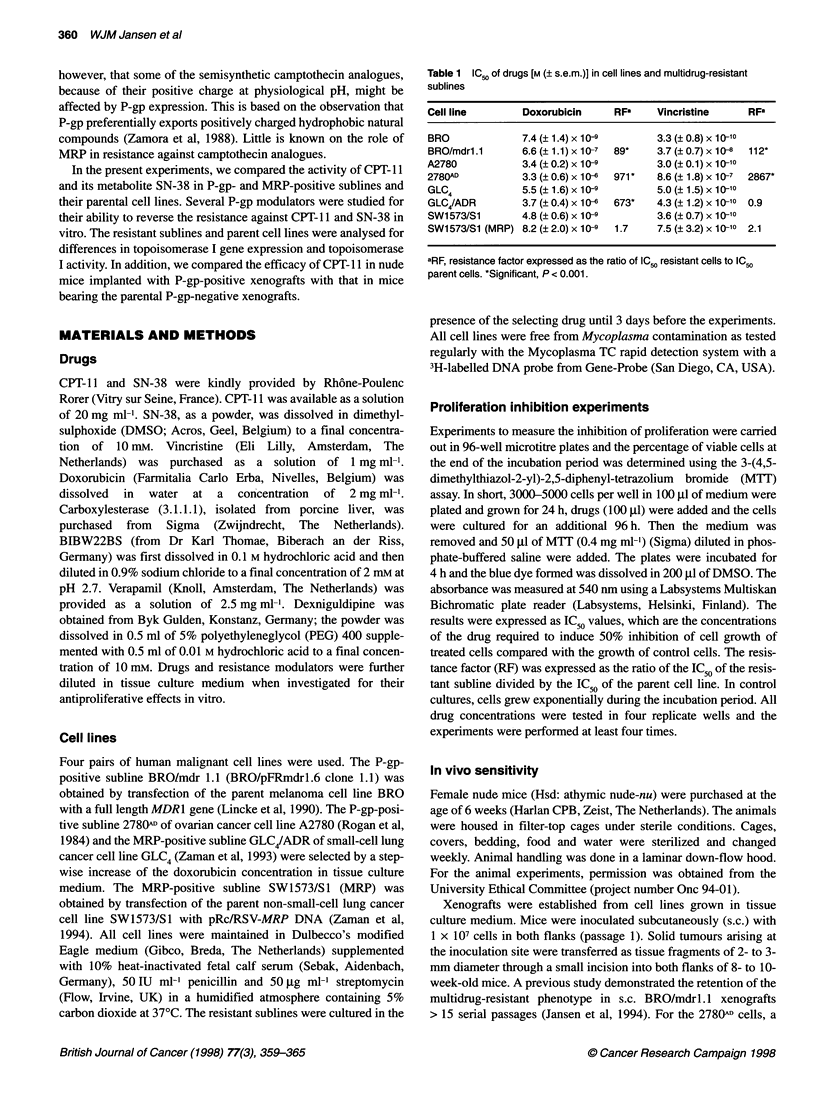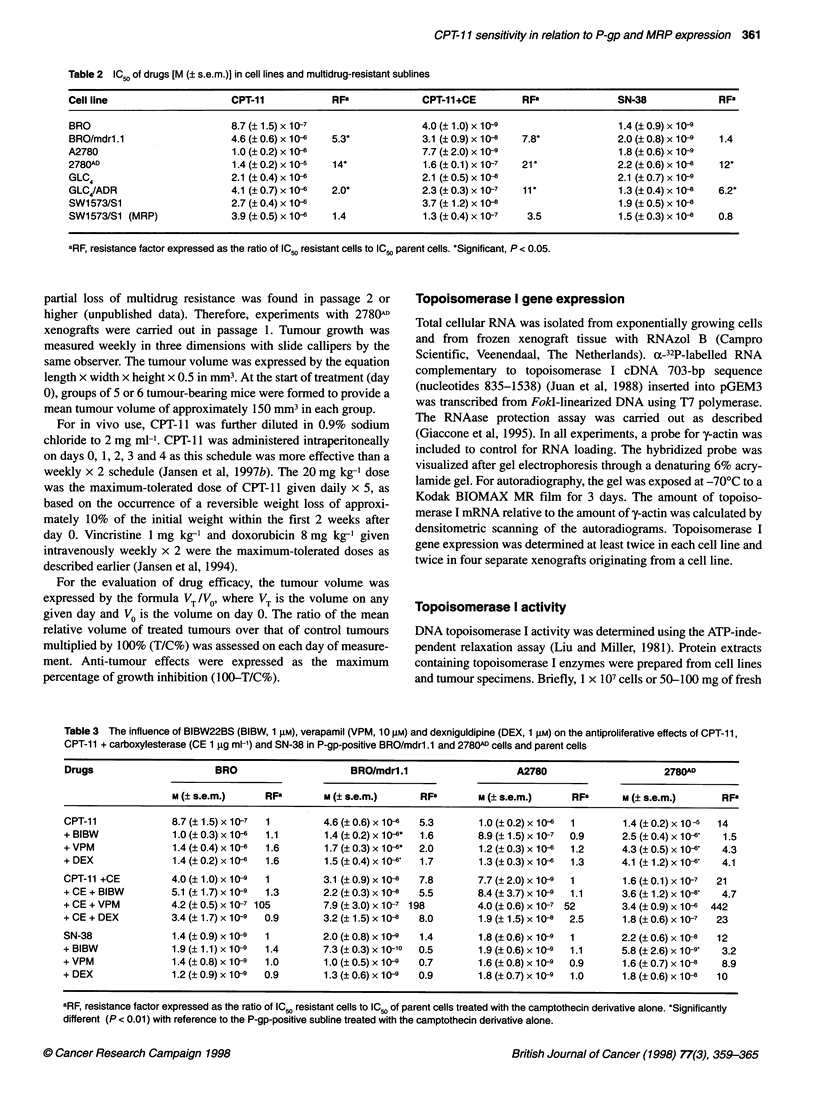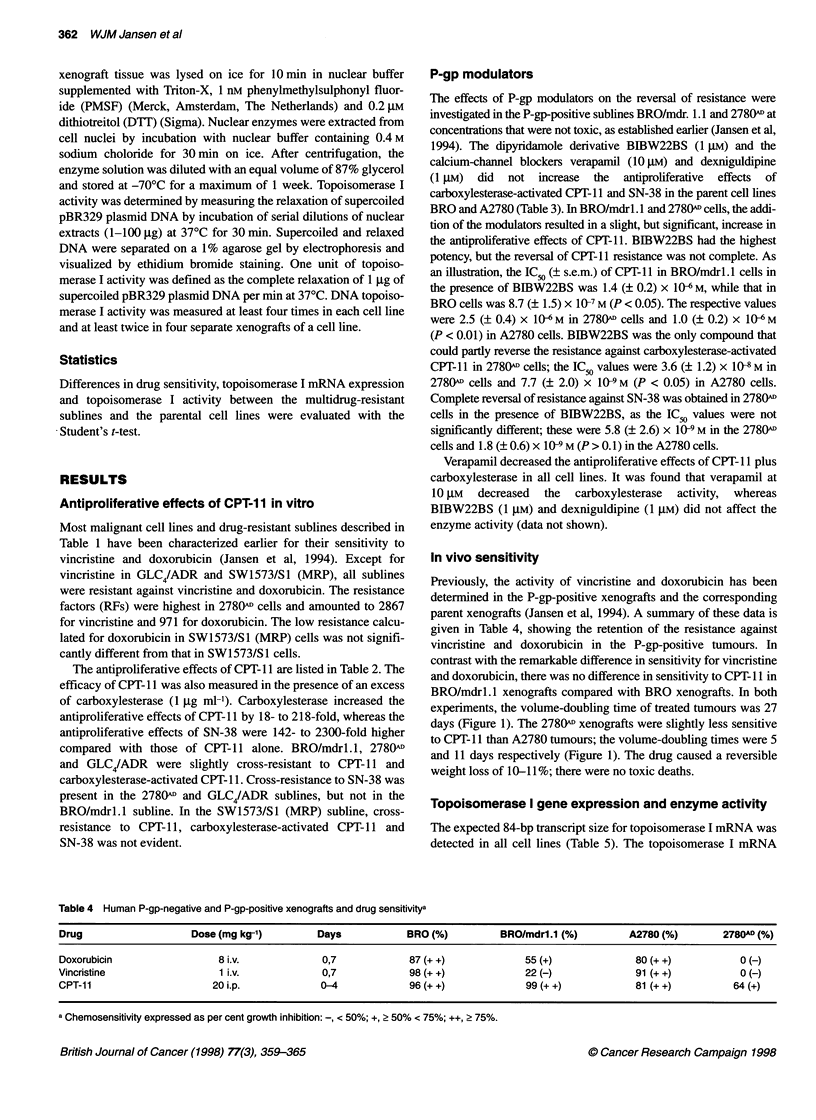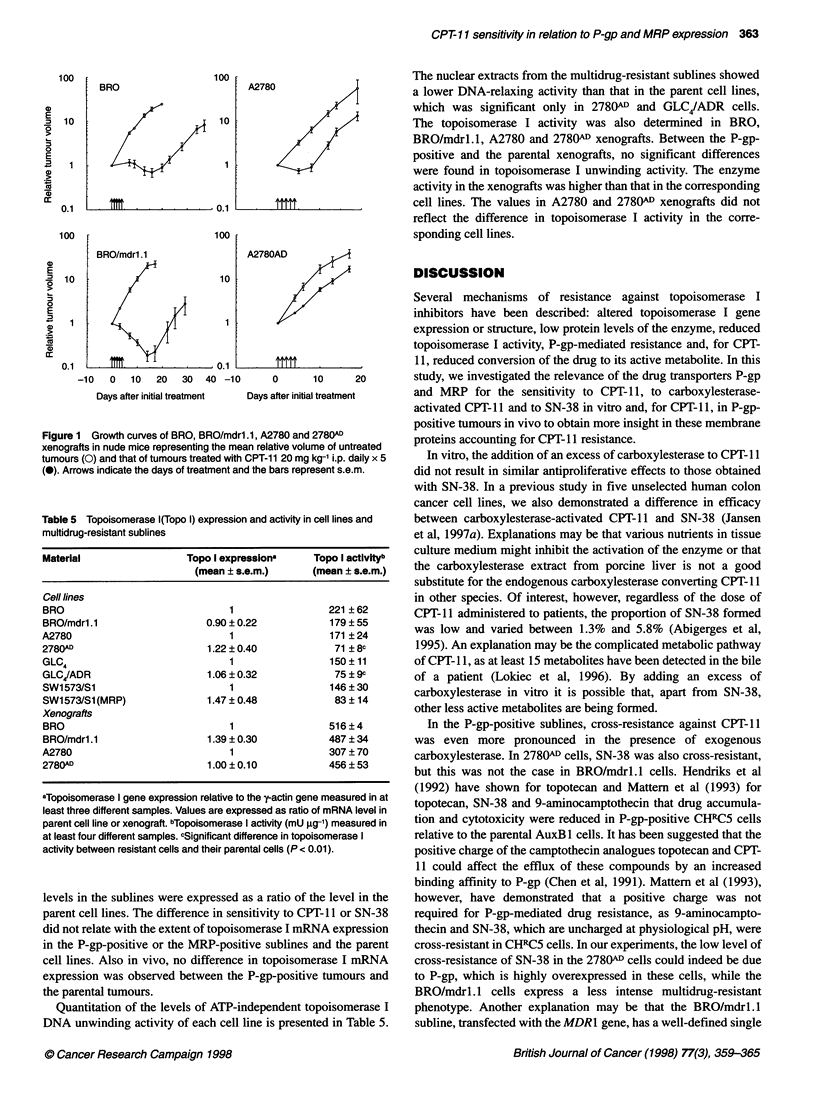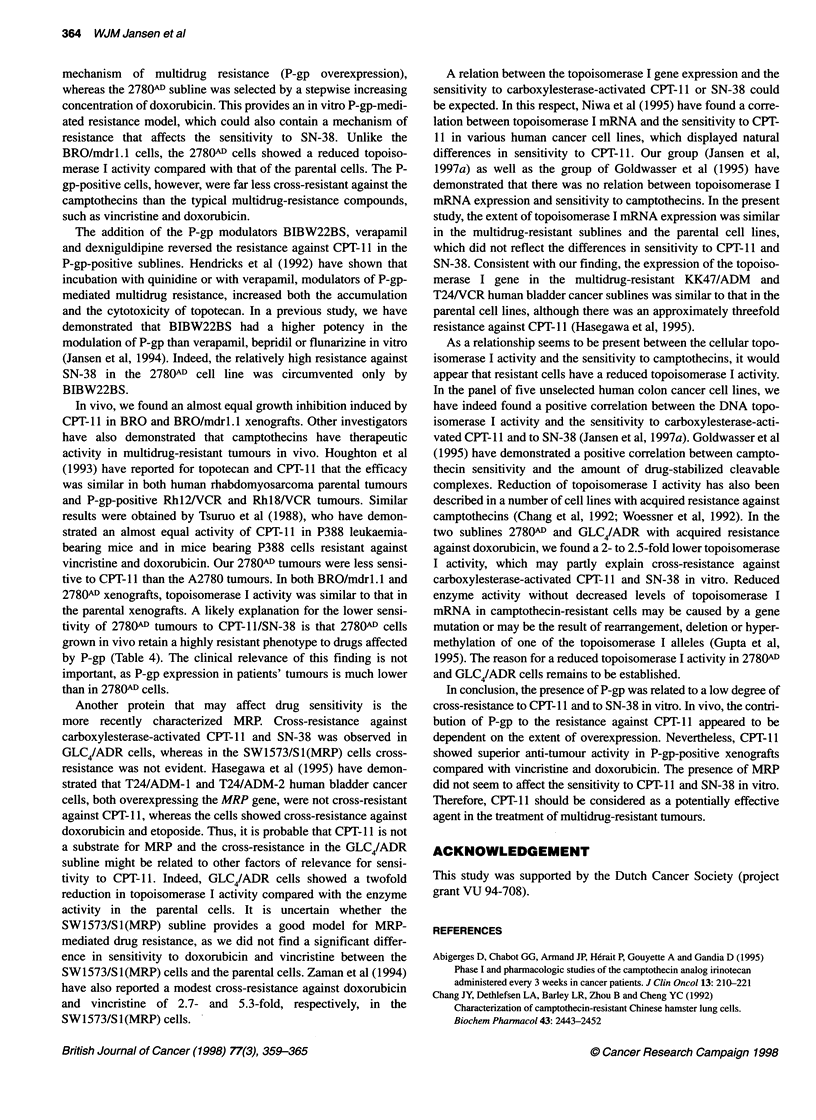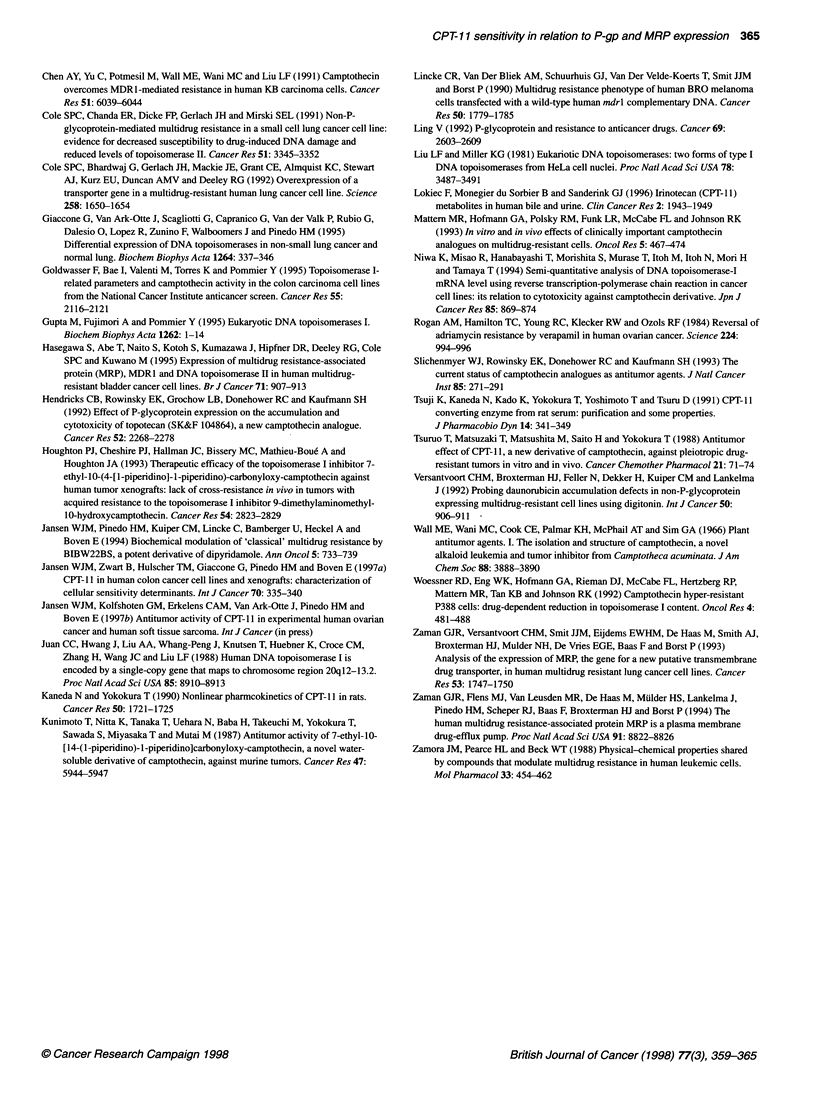# CPT-11 sensitivity in relation to the expression of P170-glycoprotein and multidrug resistance-associated protein.

**DOI:** 10.1038/bjc.1998.58

**Published:** 1998

**Authors:** W. J. Jansen, T. M. Hulscher, J. van Ark-Otte, G. Giaccone, H. M. Pinedo, E. Boven

**Affiliations:** Department of Medical Oncology, Academic Hospital Vrije Universiteit, Amsterdam, The Netherlands.

## Abstract

The relevance of P170-glycoprotein (P-gp) and multidrug resistance-associated protein (MRP) for the sensitivity to CPT-11 was investigated in human malignant cell lines as well as in human tumour xenografts. In vitro, the P-gp-positive sublines BRO/mdr1.1 (transfected with MDR1) and 2780AD were slightly cross-resistant against carboxylesterase-activated CPT-11. Cross-resistance against SN-38 was present in 2780AD cells, but not in BRO/mdr1.1 cells. The P-gp modulators BIBW22BS, verapamil and dexniguldipine partly reversed the resistance against CPT-11 in the P-gp-positive sublines. BIBW22BS was the most effective modulator in the reversal of the resistance against carboxylesterase-activated CPT-11 as well as against SN-38 in the 2780AD subline. In contrast to doxorubicin and vincristine, the BRO/mdr1.1 xenografts were at least as sensitive to CPT-11 as the BRO xenografts. The 2780AD xenografts were slightly less sensitive than the parent tumours, but there was no difference in topoisomerase I DNA unwinding activity. Therefore, the high retention of the multidrug-resistant phenotype of 2780AD cells in vivo may be the cause of the low cross-resistance against CPT-11. The MRP-positive subline GLC4/ADR was cross-resistant against carboxylesterase-activated CPT-11 and SN-38. GLC4/ADR cells, however, demonstrated a twofold lower topoisomerase I activity than GLC4 cells. Cross-resistance against the camptothecin derivatives was not apparent in the MRP-transfected subline of SW1573/S1. In conclusion, P-gp-positive cells show a low cross-resistance against CPT-11/SN38, which is only apparent with high P-gp expression in vivo. MRP does not seem to play a role in the sensitivity to CPT-11.


British Journal of Cancer (1998) 77(3), 359–365
© 1998 Cancer Research Campaign

### CPT-11 sensitivity in relation to the expression of P170-glycoprotein and multidrug resistance-associated protein

**WJM Jansen, TM Hulscher, J van Ark-Otte, G Giaccone, HM Pinedo and E Boven**

Department of Medical Oncology, Academic Hospital Vrije Universiteit, Amsterdam, The Netherlands

**Summary** The relevance of P170-glycoprotein (P-gp) and multidrug resistance-associated protein (MRP) for the sensitivity to CPT-11 was investigated in human malignant cell lines as well as in human tumour xenografts. In vitro, the P-gp-positive sublines BRO/mdr1.1 (transfected with *MDR1*) and $2780^{AD}$ were slightly cross-resistant against carboxylesterase-activated CPT-11. Cross-resistance against SN-38 was present in $2780^{AD}$ cells, but not in BRO/mdr1.1 cells. The P-gp modulators BIBW22BS, verapamil and dexniguldipine partly reversed the resistance against CPT-11 in the P-gp-positive sublines. BIBW22BS was the most effective modulator in the reversal of the resistance against carboxylesterase-activated CPT-11 as well as against SN-38 in the $2780^{AD}$ subline. In contrast to doxorubicin and vincristine, the BRO/mdr1.1 xenografts were at least as sensitive to CPT-11 as the BRO xenografts. The $2780^{AD}$ xenografts were slightly less sensitive than the parent tumours, but there was no difference in topoisomerase I DNA unwinding activity. Therefore, the high retention of the multidrug-resistant phenotype of $2780^{AD}$ cells in vivo may be the cause of the low cross-resistance against CPT-11. The MRP-positive subline $GLC_4$/ADR was cross-resistant against carboxylesterase-activated CPT-11 and SN-38. $GLC_4$/ADR cells, however, demonstrated a twofold lower topoisomerase I activity than $GLC_4$ cells. Cross-resistance against the camptothecin derivatives was not apparent in the *MRP*-transfected subline of SW1573/S1. In conclusion, P-gp-positive cells show a low cross-resistance against CPT-11/SN38, which is only apparent with high P-gp expression in vivo. MRP does not seem to play a role in the sensitivity to CPT-11.

**Keywords:** CPT-11; P170-glycoprotein; multidrug resistance-associated protein; topoisomerase I

Cancer cells treated with cytostatic agents may become cross-resistant against a range of drugs differing in structure and cell target. 'Classical' multidrug resistance is associated with increased expression of the *MDR*1 gene encoding a 170-kDa P-glycoprotein (P-gp) (Ling et al, 1992). P-gp, an ATP-dependent transport protein, is located in the plasma membrane and can extrude a range of hydrophobic natural drugs and cytostatic agents from the cancer cells against a concentration gradient. As a result, the presence of P-gp will induce drug resistance. However, another mechanism may also contribute to multidrug resistance as multidrug-resistant cells have been described that do not express P-gp (Cole et al, 1991; Versantvoort et al, 1992). In one of these non-P-gp cell lines, the H69AR small-cell lung cancer cell line, Cole et al (1992) have found amplification of a novel gene, the multidrug resistance-associated protein gene (*MRP*). Zaman et al (1994) have reported that the multidrug resistance-associated protein (MRP) also acts as a drug pump extruding hydrophobic compounds from cells against the concentration gradient. Although the drug resistance spectra associated with *MRP* and *MDR*1 overexpression are remarkably similar, there are some differences between P-gp and MRP and the drugs that they transport or interact with. Thus far, few data are available on the relevance of these proteins for resistance against the topoisomerase I-inhibiting camptothecins.

Camptothecin was isolated from the Chinese tree *Camptotheca acuminata*, as has been described by Wall et al (1966). Camptothecin shows strong anti-tumour activity against several experimental tumours. In clinical trials in the early 1970s, it failed to induce meaningful responses and proved to cause severe and unpredictable myelosuppression, haemorrhagic cystitis and diarrhoea. The interest in camptothecin was revived in the late 1980s after the identification of the enzyme topoisomerase I as the major cellular target of camptothecin (Slichenmyer et al, 1993). Much effort has been put into the synthesis of new water-soluble camptothecin derivatives with higher anti-tumour activity and less toxicity. This has led to the development of a novel water-soluble derivative 7-ethyl-10[4-(1-piperidino)-1-piperidino]carbonyloxy camptothecin (CPT-11). Although CPT-11 is very active in a wide variety of human tumour xenografts (Kunimoto et al, 1987), its in vitro activity is rather poor. CPT-11 has to be converted into 7-ethyl-10-hydroxy-camptothecin (SN-38) by a carboxylesterase to exert its action (Kaneda et al, 1990; Tsuji et al, 1991). In a number of human colon cancer cell lines SN-38 has been found to be 130- to 570-fold more active than CPT-11 (Jansen et al, 1997*a*). In the clinic, CPT-11 has substantial activity against a range of tumour types, particularly colorectal cancer, non-small-cell lung cancer and cervical cancer (Slichenmyer et al, 1993).

The efficacy of camptothecin does not seem to be affected by the presence of P-gp (Chen et al, 1991). It has been suggested,

*Received 25 February 1997*
*Revised 3 July 1997*
*Accepted 4 July 1997*

*Correspondence to:* E Boven, Department of Medical Oncology, Academic Hospital Vrije Universiteit, De Boelelaan 1117, 1081 HV Amsterdam, The Netherlands however, that some of the semisynthetic camptothecin analogues, because of their positive charge at physiological pH, might be affected by P-gp expression. This is based on the observation that P-gp preferentially exports positively charged hydrophobic natural compounds (Zamora et al, 1988). Little is known on the role of MRP in resistance against camptothecin analogues.

In the present experiments, we compared the activity of CPT-11 and its metabolite SN-38 in P-gp- and MRP-positive sublines and their parental cell lines. Several P-gp modulators were studied for their ability to reverse the resistance against CPT-11 and SN-38 in vitro. The resistant sublines and parent cell lines were analysed for differences in topoisomerase I gene expression and topoisomerase I activity. In addition, we compared the efficacy of CPT-11 in nude mice implanted with P-gp-positive xenografts with that in mice bearing the parental P-gp-negative xenografts.

### MATERIALS AND METHODS

### Drugs

CPT-11 and SN-38 were kindly provided by Rhône-Poulenc Rorer (Vitry sur Seine, France). CPT-11 was available as a solution of 20 mg ml$^{-1}$. SN-38, as a powder, was dissolved in dimethylsulphoxide (DMSO; Acros, Geel, Belgium) to a final concentration of 10 mM. Vincristine (Eli Lilly, Amsterdam, The Netherlands) was purchased as a solution of 1 mg ml$^{-1}$. Doxorubicin (Farmitalia Carlo Erba, Nivelles, Belgium) was dissolved in water at a concentration of 2 mg ml$^{-1}$. Carboxylesterase (3.1.1.1), isolated from porcine liver, was purchased from Sigma (Zwijndrecht, The Netherlands). BIBW22BS (from Dr Karl Thomae, Biberach an der Riss, Germany) was first dissolved in 0.1 M hydrochloric acid and then diluted in 0.9% sodium chloride to a final concentration of 2 mM at pH 2.7. Verapamil (Knoll, Amsterdam, The Netherlands) was provided as a solution of 2.5 mg ml$^{-1}$. Dexniguldipine was obtained from Byk Gulden, Konstanz, Germany; the powder was dissolved in 0.5 ml of 5% polyethyleneglycol (PEG) 400 supplemented with 0.5 ml of 0.01 M hydrochloric acid to a final concentration of 10 mM. Drugs and resistance modulators were further diluted in tissue culture medium when investigated for their antiproliferative effects in vitro.

### Cell lines

Four pairs of human malignant cell lines were used. The P-gp-positive subline BRO/mdr 1.1 (BRO/pFRmdr1.6 clone 1.1) was obtained by transfection of the parent melanoma cell line BRO with a full length *MDR*1 gene (Lincke et al, 1990). The P-gp-positive subline 2780$^{AD}$ of ovarian cancer cell line A2780 (Rogan et al, 1984) and the MRP-positive subline $GLC_4$/ADR of small-cell lung cancer cell line $GLC_4$ (Zaman et al, 1993) were selected by a stepwise increase of the doxorubicin concentration in tissue culture medium. The MRP-positive subline SW1573/S1 (MRP) was obtained by transfection of the parent non-small-cell lung cancer cell line SW1573/S1 with pRc/RSV-*MRP* DNA (Zaman et al, 1994). All cell lines were maintained in Dulbecco's modified Eagle medium (Gibco, Breda, The Netherlands) supplemented with 10% heat-inactivated fetal calf serum (Sebak, Aidenbach, Germany), 50 IU ml$^{-1}$ penicillin and 50 μg ml$^{-1}$ streptomycin (Flow, Irvine, UK) in a humidified atmosphere containing 5% carbon dioxide at 37°C. The resistant sublines were cultured in the presence of the selecting drug until 3 days before the experiments. All cell lines were free from *Mycoplasma* contamination as tested regularly with the Mycoplasma TC rapid detection system with a $^{3}$H-labelled DNA probe from Gene-Probe (San Diego, CA, USA).

**Table 1** $IC_{50}$ of drugs [M (± s.e.m.)] in cell lines and multidrug-resistant sublines

| Cell line | Doxorubicin | RF[a] | Vincristine | RF[a] |
|---|---|---|---|---|
| BRO | 7.4 (± 1.4) × $10^{-9}$ | | 3.3 (± 0.8) × $10^{-10}$ | |
| BRO/mdr1.1 | 6.6 (± 1.1) × $10^{-7}$ | 89* | 3.7 (± 0.7) × $10^{-8}$ | 112* |
| A2780 | 3.4 (± 0.2) × $10^{-9}$ | | 3.0 (± 0.1) × $10^{-10}$ | |
| 2780$^{AD}$ | 3.3 (± 0.6) × $10^{-6}$ | 971* | 8.6 (± 1.8) × $10^{-7}$ | 2867* |
| $GLC_4$ | 5.5 (± 1.6) × $10^{-9}$ | | 5.0 (± 1.5) × $10^{-10}$ | |
| $GLC_4$/ADR | 3.7 (± 0.4) × $10^{-6}$ | 673* | 4.3 (± 1.2) × $10^{-10}$ | 0.9 |
| SW1573/S1 | 4.8 (± 0.6) × $10^{-9}$ | | 3.6 (± 0.7) × $10^{-10}$ | |
| SW1573/S1 (MRP) | 8.2 (± 2.0) × $10^{-9}$ | 1.7 | 7.5 (± 3.2) × $10^{-10}$ | 2.1 |

[a]RF, resistance factor expressed as the ratio of $IC_{50}$ resistant cells to $IC_{50}$ parent cells. *Significant, $P < 0.001$.

### Proliferation inhibition experiments

Experiments to measure the inhibition of proliferation were carried out in 96-well microtitre plates and the percentage of viable cells at the end of the incubation period was determined using the 3-(4,5-dimethylthiazol-2-yl)-2,5-diphenyl-tetrazolium bromide (MTT) assay. In short, 3000–5000 cells per well in 100 μl of medium were plated and grown for 24 h, drugs (100 μl) were added and the cells were cultured for an additional 96 h. Then the medium was removed and 50 μl of MTT (0.4 mg ml$^{-1}$) (Sigma) diluted in phosphate-buffered saline were added. The plates were incubated for 4 h and the blue dye formed was dissolved in 200 μl of DMSO. The absorbance was measured at 540 nm using a Labsystems Multiskan Bichromatic plate reader (Labsystems, Helsinki, Finland). The results were expressed as $IC_{50}$ values, which are the concentrations of the drug required to induce 50% inhibition of cell growth of treated cells compared with the growth of control cells. The resistance factor (RF) was expressed as the ratio of the $IC_{50}$ of the resistant subline divided by the $IC_{50}$ of the parent cell line. In control cultures, cells grew exponentially during the incubation period. All drug concentrations were tested in four replicate wells and the experiments were performed at least four times.

### In vivo sensitivity

Female nude mice (Hsd: athymic nude-*nu*) were purchased at the age of 6 weeks (Harlan CPB, Zeist, The Netherlands). The animals were housed in filter-top cages under sterile conditions. Cages, covers, bedding, food and water were sterilized and changed weekly. Animal handling was done in a laminar down-flow hood. For the animal experiments, permission was obtained from the University Ethical Committee (project number Onc 94-01).

Xenografts were established from cell lines grown in tissue culture medium. Mice were inoculated subcutaneously (s.c.) with 1 × $10^7$ cells in both flanks (passage 1). Solid tumours arising at the inoculation site were transferred as tissue fragments of 2- to 3-mm diameter through a small incision into both flanks of 8- to 10-week-old mice. A previous study demonstrated the retention of the multidrug-resistant phenotype in s.c. BRO/mdr1.1 xenografts > 15 serial passages (Jansen et al, 1994). For the 2780$^{AD}$ cells, a

© Cancer Research Campaign 1998

**Table 2** $IC_{50}$ of drugs [M (± s.e.m.)] in cell lines and multidrug-resistant sublines

| Cell line | CPT-11 | RF[a] | CPT-11+CE | RF[a] | SN-38 | RF[a] |
|---|---|---|---|---|---|---|
| BRO | 8.7 (± 1.5) × $10^{-7}$ | | 4.0 (± 1.0) × $10^{-9}$ | | 1.4 (± 0.9) × $10^{-9}$ | |
| BRO/mdr1.1 | 4.6 (± 0.6) × $10^{-6}$ | 5.3* | 3.1 (± 0.9) × $10^{-8}$ | 7.8* | 2.0 (± 0.8) × $10^{-9}$ | 1.4 |
| A2780 | 1.0 (± 0.2) × $10^{-6}$ | | 7.7 (± 2.0) × $10^{-9}$ | | 1.8 (± 0.6) × $10^{-9}$ | |
| $2780^{AD}$ | 1.4 (± 0.2) × $10^{-5}$ | 14* | 1.6 (± 0.1) × $10^{-7}$ | 21* | 2.2 (± 0.6) × $10^{-8}$ | 12* |
| $GLC_4$ | 2.1 (± 0.4) × $10^{-6}$ | | 2.1 (± 0.5) × $10^{-8}$ | | 2.1 (± 0.7) × $10^{-9}$ | |
| $GLC_4$/ADR | 4.1 (± 0.7) × $10^{-6}$ | 2.0* | 2.3 (± 0.3) × $10^{-7}$ | 11* | 1.3 (± 0.4) × $10^{-8}$ | 6.2* |
| SW1573/S1 | 2.7 (± 0.4) × $10^{-6}$ | | 3.7 (± 1.2) × $10^{-8}$ | | 1.9 (± 0.5) × $10^{-8}$ | |
| SW1573/S1 (MRP) | 3.9 (± 0.5) × $10^{-6}$ | 1.4 | 1.3 (± 0.4) × $10^{-7}$ | 3.5 | 1.5 (± 0.3) × $10^{-8}$ | 0.8 |

[a]RF, resistance factor expressed as the ratio of $IC_{50}$ resistant cells to $IC_{50}$ parent cells. *Significant, $P < 0.05$.

partial loss of multidrug resistance was found in passage 2 or higher (unpublished data). Therefore, experiments with $2780^{AD}$ xenografts were carried out in passage 1. Tumour growth was measured weekly in three dimensions with slide callipers by the same observer. The tumour volume was expressed by the equation length × width × height × 0.5 in $mm^3$. At the start of treatment (day 0), groups of 5 or 6 tumour-bearing mice were formed to provide a mean tumour volume of approximately 150 $mm^3$ in each group.

For in vivo use, CPT-11 was further diluted in 0.9% sodium chloride to 2 mg $ml^{-1}$. CPT-11 was administered intraperitoneally on days 0, 1, 2, 3 and 4 as this schedule was more effective than a weekly × 2 schedule (Jansen et al, 1997*b*). The 20 mg $kg^{-1}$ dose was the maximum-tolerated dose of CPT-11 given daily × 5, as based on the occurrence of a reversible weight loss of approximately 10% of the initial weight within the first 2 weeks after day 0. Vincristine 1 mg $kg^{-1}$ and doxorubicin 8 mg $kg^{-1}$ given intravenously weekly × 2 were the maximum-tolerated doses as described earlier (Jansen et al, 1994).

For the evaluation of drug efficacy, the tumour volume was expressed by the formula $V_T/V_0$, where $V_T$ is the volume on any given day and $V_0$ is the volume on day 0. The ratio of the mean relative volume of treated tumours over that of control tumours multiplied by 100% (T/C%) was assessed on each day of measurement. Anti-tumour effects were expressed as the maximum percentage of growth inhibition (100–T/C%).

### Topoisomerase I gene expression

Total cellular RNA was isolated from exponentially growing cells and from frozen xenograft tissue with RNAzol B (Campro Scientific, Veenendaal, The Netherlands). $\alpha$-$^{32}$P-labelled RNA complementary to topoisomerase I cDNA 703-bp sequence (nucleotides 835–1538) (Juan et al, 1988) inserted into pGEM3 was transcribed from *Fok*I-linearized DNA using T7 polymerase. The RNAase protection assay was carried out as described (Giaccone et al, 1995). In all experiments, a probe for $\gamma$-actin was included to control for RNA loading. The hybridized probe was visualized after gel electrophoresis through a denaturing 6% acrylamide gel. For autoradiography, the gel was exposed at –70°C to a Kodak BIOMAX MR film for 3 days. The amount of topoisomerase I mRNA relative to the amount of $\gamma$-actin was calculated by densitometric scanning of the autoradiograms. Topoisomerase I gene expression was determined at least twice in each cell line and twice in four separate xenografts originating from a cell line.

### Topoisomerase I activity

DNA topoisomerase I activity was determined using the ATP-independent relaxation assay (Liu and Miller, 1981). Protein extracts containing topoisomerase I enzymes were prepared from cell lines and tumour specimens. Briefly, 1 × $10^7$ cells or 50–100 mg of fresh

**Table 3** The influence of BIBW22BS (BIBW, 1 µM), verapamil (VPM, 10 µM) and dexniguldipine (DEX, 1 µM) on the antiproliferative effects of CPT-11, CPT-11 + carboxylesterase (CE 1 µg $ml^{-1}$) and SN-38 in P-gp-positive BRO/mdr1.1 and $2780^{AD}$ cells and parent cells

| Drugs | BRO | | BRO/mdr1.1 | | A2780 | | $2780^{AD}$ | |
|---|---|---|---|---|---|---|---|---|
| | M (± s.e.m.) | RF[a] | M (± s.e.m.) | RF[a] | M (± s.e.m.) | RF[a] | M (± s.e.m.) | RF[a] |
| CPT-11 | 8.7 (± 1.5) × $10^{-7}$ | 1 | 4.6 (± 0.6) × $10^{-6}$ | 5.3 | 1.0 (± 0.2) × $10^{-6}$ | 1 | 1.4 (± 0.2) × $10^{-5}$ | 14 |
| + BIBW | 1.0 (± 0.3) × $10^{-6}$ | 1.1 | 1.4 (± 0.2) × $10^{-6}$* | 1.6 | 8.9 (± 1.5) × $10^{-7}$ | 0.9 | 2.5 (± 0.4) × $10^{-6}$* | 1.5 |
| + VPM | 1.4 (± 0.4) × $10^{-6}$ | 1.6 | 1.7 (± 0.3) × $10^{-6}$* | 2.0 | 1.2 (± 0.3) × $10^{-6}$ | 1.2 | 4.3 (± 0.5) × $10^{-6}$* | 4.3 |
| + DEX | 1.4 (± 0.2) × $10^{-6}$ | 1.6 | 1.5 (± 0.4) × $10^{-6}$* | 1.7 | 1.3 (± 0.3) × $10^{-6}$ | 1.3 | 4.1 (± 1.2) × $10^{-6}$* | 4.1 |
| CPT-11 +CE | 4.0 (± 1.0) × $10^{-9}$ | 1 | 3.1 (± 0.9) × $10^{-8}$ | 7.8 | 7.7 (± 2.0) × $10^{-9}$ | 1 | 1.6 (± 0.1) × $10^{-7}$ | 21 |
| + CE + BIBW | 5.1 (± 1.7) × $10^{-9}$ | 1.3 | 2.2 (± 0.3) × $10^{-8}$ | 5.5 | 8.4 (± 3.7) × $10^{-9}$ | 1.1 | 3.6 (± 1.2) × $10^{-8}$* | 4.7 |
| + CE + VPM | 4.2 (± 0.5) × $10^{-7}$ | 105 | 7.9 (± 3.0) × $10^{-7}$ | 198 | 4.0 (± 0.6) × $10^{-7}$ | 52 | 3.4 (± 0.9) × $10^{-6}$ | 442 |
| + CE + DEX | 3.4 (± 1.7) × $10^{-9}$ | 0.9 | 3.2 (± 1.5) × $10^{-8}$ | 8.0 | 1.9 (± 1.5) × $10^{-8}$ | 2.5 | 1.8 (± 0.6) × $10^{-7}$ | 23 |
| SN-38 | 1.4 (± 0.9) × $10^{-9}$ | 1 | 2.0 (± 0.8) × $10^{-9}$ | 1.4 | 1.8 (± 0.6) × $10^{-9}$ | 1 | 2.2 (± 0.6) × $10^{-8}$ | 12 |
| + BIBW | 1.9 (± 1.1) × $10^{-9}$ | 1.4 | 7.3 (± 0.3) × $10^{-10}$ | 0.5 | 1.9 (± 0.6) × $10^{-9}$ | 1.1 | 5.8 (± 2.6) × $10^{-9}$* | 3.2 |
| + VPM | 1.4 (± 0.8) × $10^{-9}$ | 1.0 | 1.0 (± 0.5) × $10^{-9}$ | 0.7 | 1.6 (± 0.8) × $10^{-9}$ | 0.9 | 1.6 (± 0.7) × $10^{-8}$ | 8.9 |
| + DEX | 1.2 (± 0.9) × $10^{-9}$ | 0.9 | 1.3 (± 0.6) × $10^{-9}$ | 0.9 | 1.8 (± 0.7) × $10^{-9}$ | 1.0 | 1.8 (± 0.6) × $10^{-8}$ | 10 |

[a]RF, resistance factor expressed as the ratio of $IC_{50}$ resistant cells to $IC_{50}$ of parent cells treated with the camptothecin derivative alone. *Significantly different ($P < 0.01$) with reference to the P-gp-positive subline treated with the camptothecin derivative alone.

© Cancer Research Campaign 1998

xenograft tissue was lysed on ice for 10 min in nuclear buffer supplemented with Triton-X, 1 nM phenylmethylsulphonyl fluoride (PMSF) (Merck, Amsterdam, The Netherlands) and 0.2 μM dithiotreitol (DTT) (Sigma). Nuclear enzymes were extracted from cell nuclei by incubation with nuclear buffer containing 0.4 M sodium choloride for 30 min on ice. After centrifugation, the enzyme solution was diluted with an equal volume of 87% glycerol and stored at –70°C for a maximum of 1 week. Topoisomerase I activity was determined by measuring the relaxation of supercoiled pBR329 plasmid DNA by incubation of serial dilutions of nuclear extracts (1–100 μg) at 37°C for 30 min. Supercoiled and relaxed DNA were separated on a 1% agarose gel by electrophoresis and visualized by ethidium bromide staining. One unit of topoisomerase I activity was defined as the complete relaxation of 1 μg of supercoiled pBR329 plasmid DNA per min at 37°C. DNA topoisomerase I activity was measured at least four times in each cell line and at least twice in four separate xenografts of a cell line.

### Statistics

Differences in drug sensitivity, topoisomerase I mRNA expression and topoisomerase I activity between the multidrug-resistant sublines and the parental cell lines were evaluated with the Student's *t*-test.

### RESULTS

### Antiproliferative effects of CPT-11 in vitro

Most malignant cell lines and drug-resistant sublines described in Table 1 have been characterized earlier for their sensitivity to vincristine and doxorubicin (Jansen et al, 1994). Except for vincristine in $GLC_4$/ADR and SW1573/S1 (MRP), all sublines were resistant against vincristine and doxorubicin. The resistance factors (RFs) were highest in $2780^{AD}$ cells and amounted to 2867 for vincristine and 971 for doxorubicin. The low resistance calculated for doxorubicin in SW1573/S1 (MRP) cells was not significantly different from that in SW1573/S1 cells.

The antiproliferative effects of CPT-11 are listed in Table 2. The efficacy of CPT-11 was also measured in the presence of an excess of carboxylesterase (1 μg $ml^{-1}$). Carboxylesterase increased the antiproliferative effects of CPT-11 by 18- to 218-fold, whereas the antiproliferative effects of SN-38 were 142- to 2300-fold higher compared with those of CPT-11 alone. BRO/mdr1.1, $2780^{AD}$ and $GLC_4$/ADR were slightly cross-resistant to CPT-11 and carboxylesterase-activated CPT-11. Cross-resistance to SN-38 was present in the $2780^{AD}$ and $GLC_4$/ADR sublines, but not in the BRO/mdr1.1 subline. In the SW1573/S1 (MRP) subline, cross-resistance to CPT-11, carboxylesterase-activated CPT-11 and SN-38 was not evident.

### P-gp modulators

The effects of P-gp modulators on the reversal of resistance were investigated in the P-gp-positive sublines BRO/mdr. 1.1 and $2780^{AD}$ at concentrations that were not toxic, as established earlier (Jansen et al, 1994). The dipyridamole derivative BIBW22BS (1 μM) and the calcium-channel blockers verapamil (10 μM) and dexniguldipine (1 μM) did not increase the antiproliferative effects of carboxylesterase-activated CPT-11 and SN-38 in the parent cell lines BRO and A2780 (Table 3). In BRO/mdr1.1 and $2780^{AD}$ cells, the addition of the modulators resulted in a slight, but significant, increase in the antiproliferative effects of CPT-11. BIBW22BS had the highest potency, but the reversal of CPT-11 resistance was not complete. As an illustration, the $IC_{50}$ (± s.e.m.) of CPT-11 in BRO/mdr1.1 cells in the presence of BIBW22BS was 1.4 (± 0.2) × $10^{-6}$ M, while that in BRO cells was 8.7 (± 1.5) × $10^{-7}$ M ($P < 0.05$). The respective values were 2.5 (± 0.4) × $10^{-6}$ M in $2780^{AD}$ cells and 1.0 (± 0.2) × $10^{-6}$ M ($P < 0.01$) in A2780 cells. BIBW22BS was the only compound that could partly reverse the resistance against carboxylesterase-activated CPT-11 in $2780^{AD}$ cells; the $IC_{50}$ values were 3.6 (± 1.2) × $10^{-8}$ M in $2780^{AD}$ cells and 7.7 (± 2.0) × $10^{-9}$ M ($P < 0.05$) in A2780 cells. Complete reversal of resistance against SN-38 was obtained in $2780^{AD}$ cells in the presence of BIBW22BS, as the $IC_{50}$ values were not significantly different; these were 5.8 (± 2.6) × $10^{-9}$ M in the $2780^{AD}$ cells and 1.8 (± 0.6) × $10^{-9}$ M ($P > 0.1$) in the A2780 cells.

Verapamil decreased the antiproliferative effects of CPT-11 plus carboxylesterase in all cell lines. It was found that verapamil at 10 μM decreased the carboxylesterase activity, whereas BIBW22BS (1 μM) and dexniguldipine (1 μM) did not affect the enzyme activity (data not shown).

### In vivo sensitivity

Previously, the activity of vincristine and doxorubicin has been determined in the P-gp-positive xenografts and the corresponding parent xenografts (Jansen et al, 1994). A summary of these data is given in Table 4, showing the retention of the resistance against vincristine and doxorubicin in the P-gp-positive tumours. In contrast with the remarkable difference in sensitivity for vincristine and doxorubicin, there was no difference in sensitivity to CPT-11 in BRO/mdr1.1 xenografts compared with BRO xenografts. In both experiments, the volume-doubling time of treated tumours was 27 days (Figure 1). The $2780^{AD}$ xenografts were slightly less sensitive to CPT-11 than A2780 tumours; the volume-doubling times were 5 and 11 days respectively (Figure 1). The drug caused a reversible weight loss of 10–11%; there were no toxic deaths.

### Topoisomerase I gene expression and enzyme activity

The expected 84-bp transcript size for topoisomerase I mRNA was detected in all cell lines (Table 5). The topoisomerase I mRNA

**Table 4** Human P-gp-negative and P-gp-positive xenografts and drug sensitivity[a]

| Drug | Dose (mg $kg^{-1}$) | Days | BRO (%) | BRO/mdr1.1 (%) | A2780 (%) | $2780^{AD}$ (%) |
|---|---|---|---|---|---|---|
| Doxorubicin | 8 i.v. | 0,7 | 87 (+ +) | 55 (+) | 80 (+ +) | 0 (–) |
| Vincristine | 1 i.v. | 0,7 | 98 (+ +) | 22 (–) | 91 (+ +) | 0 (–) |
| CPT-11 | 20 i.p. | 0–4 | 96 (+ +) | 99 (+ +) | 81 (+ +) | 64 (+) |

[a] Chemosensitivity expressed as per cent growth inhibition: –, < 50%; +, ≥ 50% < 75%; ++, ≥ 75%.

© Cancer Research Campaign 1998

**Figure 1** Growth curves of BRO, BRO/mdr1.1, A2780 and $2780^{AD}$ xenografts in nude mice representing the mean relative volume of untreated tumours (○) and that of tumours treated with CPT-11 20 mg kg$^{-1}$ i.p. daily × 5 (●). Arrows indicate the days of treatment and the bars represent s.e.m.

**Table 5** Topoisomerase I(Topo I) expression and activity in cell lines and multidrug-resistant sublines

| Material | Topo I expression[a] (mean ± s.e.m.) | Topo I activity[b] (mean ± s.e.m.) |
|---|---|---|
| *Cell lines* | | |
| BRO | 1 | 221 ± 62 |
| BRO/mdr1.1 | 0.90 ± 0.22 | 179 ± 55 |
| A2780 | 1 | 171 ± 24 |
| $2780^{AD}$ | 1.22 ± 0.40 | 71 ± 8[c] |
| $GLC_4$ | 1 | 150 ± 11 |
| $GLC_4$/ADR | 1.06 ± 0.32 | 75 ± 9[c] |
| SW1573/S1 | 1 | 146 ± 30 |
| SW1573/S1(MRP) | 1.47 ± 0.48 | 83 ± 14 |
| *Xenografts* | | |
| BRO | 1 | 516 ± 4 |
| BRO/mdr1.1 | 1.39 ± 0.30 | 487 ± 34 |
| A2780 | 1 | 307 ± 70 |
| $2780^{AD}$ | 1.00 ± 0.10 | 456 ± 53 |

[a]Topoisomerase I gene expression relative to the γ-actin gene measured in at least three different samples. Values are expressed as ratio of mRNA level in parent cell line or xenograft. [b]Topoisomerase I activity (mU µg$^{-1}$) measured in at least four different samples. [c]Significant difference in topoisomerase I activity between resistant cells and their parental cells ($P < 0.01$).

levels in the sublines were expressed as a ratio of the level in the parent cell lines. The difference in sensitivity to CPT-11 or SN-38 did not relate with the extent of topoisomerase I mRNA expression in the P-gp-positive or the MRP-positive sublines and the parent cell lines. Also in vivo, no difference in topoisomerase I mRNA expression was observed between the P-gp-positive tumours and the parental tumours.

Quantitation of the levels of ATP-independent topoisomerase I DNA unwinding activity of each cell line is presented in Table 5. The nuclear extracts from the multidrug-resistant sublines showed a lower DNA-relaxing activity than that in the parent cell lines, which was significant only in $2780^{AD}$ and $GLC_4$/ADR cells. The topoisomerase I activity was also determined in BRO, BRO/mdr1.1, A2780 and $2780^{AD}$ xenografts. Between the P-gp-positive and the parental xenografts, no significant differences were found in topoisomerase I unwinding activity. The enzyme activity in the xenografts was higher than that in the corresponding cell lines. The values in A2780 and $2780^{AD}$ xenografts did not reflect the difference in topoisomerase I activity in the corresponding cell lines.

### DISCUSSION

Several mechanisms of resistance against topoisomerase I inhibitors have been described: altered topoisomerase I gene expression or structure, low protein levels of the enzyme, reduced topoisomerase I activity, P-gp-mediated resistance and, for CPT-11, reduced conversion of the drug to its active metabolite. In this study, we investigated the relevance of the drug transporters P-gp and MRP for the sensitivity to CPT-11, to carboxylesterase-activated CPT-11 and to SN-38 in vitro and, for CPT-11, in P-gp-positive tumours in vivo to obtain more insight in these membrane proteins accounting for CPT-11 resistance.

In vitro, the addition of an excess of carboxylesterase to CPT-11 did not result in similar antiproliferative effects to those obtained with SN-38. In a previous study in five unselected human colon cancer cell lines, we also demonstrated a difference in efficacy between carboxylesterase-activated CPT-11 and SN-38 (Jansen et al, 1997*a*). Explanations may be that various nutrients in tissue culture medium might inhibit the activation of the enzyme or that the carboxylesterase extract from porcine liver is not a good substitute for the endogenous carboxylesterase converting CPT-11 in other species. Of interest, however, regardless of the dose of CPT-11 administered to patients, the proportion of SN-38 formed was low and varied between 1.3% and 5.8% (Abigerges et al, 1995). An explanation may be the complicated metabolic pathway of CPT-11, as at least 15 metabolites have been detected in the bile of a patient (Lokiec et al, 1996). By adding an excess of carboxylesterase in vitro it is possible that, apart from SN-38, other less active metabolites are being formed.

In the P-gp-positive sublines, cross-resistance against CPT-11 was even more pronounced in the presence of exogenous carboxylesterase. In $2780^{AD}$ cells, SN-38 was also cross-resistant, but this was not the case in BRO/mdr1.1 cells. Hendriks et al (1992) have shown for topotecan and Mattern et al (1993) for topotecan, SN-38 and 9-aminocamptothecin that drug accumulation and cytotoxicity were reduced in P-gp-positive $CH^RC5$ cells relative to the parental AuxB1 cells. It has been suggested that the positive charge of the camptothecin analogues topotecan and CPT-11 could affect the efflux of these compounds by an increased binding affinity to P-gp (Chen et al, 1991). Mattern et al (1993), however, have demonstrated that a positive charge was not required for P-gp-mediated drug resistance, as 9-aminocamptothecin and SN-38, which are uncharged at physiological pH, were cross-resistant in $CH^RC5$ cells. In our experiments, the low level of cross-resistance of SN-38 in the $2780^{AD}$ cells could indeed be due to P-gp, which is highly overexpressed in these cells, while the BRO/mdr1.1 cells express a less intense multidrug-resistant phenotype. Another explanation may be that the BRO/mdr1.1 subline, transfected with the *MDR*1 gene, has a well-defined single

© Cancer Research Campaign 1998

mechanism of multidrug resistance (P-gp overexpression), whereas the $2780^{AD}$ subline was selected by a stepwise increasing concentration of doxorubicin. This provides an in vitro P-gp-mediated resistance model, which could also contain a mechanism of resistance that affects the sensitivity to SN-38. Unlike the BRO/mdr1.1 cells, the $2780^{AD}$ cells showed a reduced topoisomerase I activity compared with that of the parental cells. The P-gp-positive cells, however, were far less cross-resistant against the camptothecins than the typical multidrug-resistance compounds, such as vincristine and doxorubicin.

The addition of the P-gp modulators BIBW22BS, verapamil and dexniguldipine reversed the resistance against CPT-11 in the P-gp-positive sublines. Hendricks et al (1992) have shown that incubation with quinidine or with verapamil, modulators of P-gp-mediated multidrug resistance, increased both the accumulation and the cytotoxicity of topotecan. In a previous study, we have demonstrated that BIBW22BS had a higher potency in the modulation of P-gp than verapamil, bepridil or flunarizine in vitro (Jansen et al, 1994). Indeed, the relatively high resistance against SN-38 in the $2780^{AD}$ cell line was circumvented only by BIBW22BS.

In vivo, we found an almost equal growth inhibition induced by CPT-11 in BRO and BRO/mdr1.1 xenografts. Other investigators have also demonstrated that camptothecins have therapeutic activity in multidrug-resistant tumours in vivo. Houghton et al (1993) have reported for topotecan and CPT-11 that the efficacy was similar in both human rhabdomyosarcoma parental tumours and P-gp-positive Rh12/VCR and Rh18/VCR tumours. Similar results were obtained by Tsuruo et al (1988), who have demonstrated an almost equal activity of CPT-11 in P388 leukaemia-bearing mice and in mice bearing P388 cells resistant against vincristine and doxorubicin. Our $2780^{AD}$ tumours were less sensitive to CPT-11 than the A2780 tumours. In both BRO/mdr1.1 and $2780^{AD}$ xenografts, topoisomerase I activity was similar to that in the parental xenografts. A likely explanation for the lower sensitivity of $2780^{AD}$ tumours to CPT-11/SN-38 is that $2780^{AD}$ cells grown in vivo retain a highly resistant phenotype to drugs affected by P-gp (Table 4). The clinical relevance of this finding is not important, as P-gp expression in patients' tumours is much lower than in $2780^{AD}$ cells.

Another protein that may affect drug sensitivity is the more recently characterized MRP. Cross-resistance against carboxylesterase-activated CPT-11 and SN-38 was observed in $GLC_4$/ADR cells, whereas in the SW1573/S1(MRP) cells cross-resistance was not evident. Hasegawa et al (1995) have demonstrated that T24/ADM-1 and T24/ADM-2 human bladder cancer cells, both overexpressing the *MRP* gene, were not cross-resistant against CPT-11, whereas the cells showed cross-resistance against doxorubicin and etoposide. Thus, it is probable that CPT-11 is not a substrate for MRP and the cross-resistance in the $GLC_4$/ADR subline might be related to other factors of relevance for sensitivity to CPT-11. Indeed, $GLC_4$/ADR cells showed a twofold reduction in topoisomerase I activity compared with the enzyme activity in the parental cells. It is uncertain whether the SW1573/S1(MRP) subline provides a good model for MRP-mediated drug resistance, as we did not find a significant difference in sensitivity to doxorubicin and vincristine between the SW1573/S1(MRP) cells and the parental cells. Zaman et al (1994) have also reported a modest cross-resistance against doxorubicin and vincristine of 2.7- and 5.3-fold, respectively, in the SW1573/S1(MRP) cells.

A relation between the topoisomerase I gene expression and the sensitivity to carboxylesterase-activated CPT-11 or SN-38 could be expected. In this respect, Niwa et al (1995) have found a correlation between topoisomerase I mRNA and the sensitivity to CPT-11 in various human cancer cell lines, which displayed natural differences in sensitivity to CPT-11. Our group (Jansen et al, 1997*a*) as well as the group of Goldwasser et al (1995) have demonstrated that there was no relation between topoisomerase I mRNA expression and sensitivity to camptothecins. In the present study, the extent of topoisomerase I mRNA expression was similar in the multidrug-resistant sublines and the parental cell lines, which did not reflect the differences in sensitivity to CPT-11 and SN-38. Consistent with our finding, the expression of the topoisomerase I gene in the multidrug-resistant KK47/ADM and T24/VCR human bladder cancer sublines was similar to that in the parental cell lines, although there was an approximately threefold resistance against CPT-11 (Hasegawa et al, 1995).

As a relationship seems to be present between the cellular topoisomerase I activity and the sensitivity to camptothecins, it would appear that resistant cells have a reduced topoisomerase I activity. In the panel of five unselected human colon cancer cell lines, we have indeed found a positive correlation between the DNA topoisomerase I activity and the sensitivity to carboxylesterase-activated CPT-11 and to SN-38 (Jansen et al, 1997*a*). Goldwasser et al (1995) have demonstrated a positive correlation between camptothecin sensitivity and the amount of drug-stabilized cleavable complexes. Reduction of topoisomerase I activity has also been described in a number of cell lines with acquired resistance against camptothecins (Chang et al, 1992; Woessner et al, 1992). In the two sublines $2780^{AD}$ and $GLC_4$/ADR with acquired resistance against doxorubicin, we found a 2- to 2.5-fold lower topoisomerase I activity, which may partly explain cross-resistance against carboxylesterase-activated CPT-11 and SN-38 in vitro. Reduced enzyme activity without decreased levels of topoisomerase I mRNA in camptothecin-resistant cells may be caused by a gene mutation or may be the result of rearrangement, deletion or hypermethylation of one of the topoisomerase I alleles (Gupta et al, 1995). The reason for a reduced topoisomerase I activity in $2780^{AD}$ and $GLC_4$/ADR cells remains to be established.

In conclusion, the presence of P-gp was related to a low degree of cross-resistance to CPT-11 and to SN-38 in vitro. In vivo, the contribution of P-gp to the resistance against CPT-11 appeared to be dependent on the extent of overexpression. Nevertheless, CPT-11 showed superior anti-tumour activity in P-gp-positive xenografts compared with vincristine and doxorubicin. The presence of MRP did not seem to affect the sensitivity to CPT-11 and SN-38 in vitro. Therefore, CPT-11 should be considered as a potentially effective agent in the treatment of multidrug-resistant tumours.

### ACKNOWLEDGEMENT

This study was supported by the Dutch Cancer Society (project grant VU 94-708).

### REFERENCES

Abigerges D, Chabot GG, Armand JP, Hérait P, Gouyette A and Gandia D (1995) Phase I and pharmacologic studies of the camptothecin analog irinotecan administered every 3 weeks in cancer patients. *J Clin Oncol* **13**: 210–221

Chang JY, Dethlefsen LA, Barley LR, Zhou B and Cheng YC (1992) Characterization of camptothecin-resistant Chinese hamster lung cells. *Biochem Pharmacol* **43**: 2443–2452

© Cancer Research Campaign 1998

Chen AY, Yu C, Potmesil M, Wall ME, Wani MC and Liu LF (1991) Camptothecin overcomes MDR1-mediated resistance in human KB carcinoma cells. *Cancer Res* **51**: 6039–6044

Cole SPC, Chanda ER, Dicke FP, Gerlach JH and Mirski SEL (1991) Non-P-glycoprotein-mediated multidrug resistance in a small cell lung cancer cell line: evidence for decreased susceptibility to drug-induced DNA damage and reduced levels of topoisomerase II. *Cancer Res* **51**: 3345–3352

Cole SPC, Bhardwaj G, Gerlach JH, Mackie JE, Grant CE, Almquist KC, Stewart AJ, Kurz EU, Duncan AMV and Deeley RG (1992) Overexpression of a transporter gene in a multidrug-resistant human lung cancer cell line. *Science* **258**: 1650–1654

Giaccone G, Van Ark-Otte J, Scagliotti G, Capranico G, Van der Valk P, Rubio G, Dalesio O, Lopez R, Zunino F, Walboomers J and Pinedo HM (1995) Differential expression of DNA topoisomerases in non-small lung cancer and normal lung. *Biochem Biophys Acta* **1264**: 337–346

Goldwasser F, Bae I, Valenti M, Torres K and Pommier Y (1995) Topoisomerase I-related parameters and camptothecin activity in the colon carcinoma cell lines from the National Cancer Institute anticancer screen. *Cancer Res* **55**: 2116–2121

Gupta M, Fujimori A and Pommier Y (1995) Eukaryotic DNA topoisomerases I. *Biochem Biophys Acta* **1262**: 1–14

Hasegawa S, Abe T, Naito S, Kotoh S, Kumazawa J, Hipfner DR, Deeley RG, Cole SPC and Kuwano M (1995) Expression of multidrug resistance-associated protein (MRP), MDR1 and DNA topoisomerase II in human multidrug-resistant bladder cancer cell lines. *Br J Cancer* **71**: 907–913

Hendricks CB, Rowinsky EK, Grochow LB, Donehower RC and Kaufmann SH (1992) Effect of P-glycoprotein expression on the accumulation and cytotoxicity of topotecan (SK&F 104864), a new camptothecin analogue. *Cancer Res* **52**: 2268–2278

Houghton PJ, Cheshire PJ, Hallman JC, Bissery MC, Mathieu-Boué A and Houghton JA (1993) Therapeutic efficacy of the topoisomerase I inhibitor 7-ethyl-10-(4-[1-piperidino]-1-piperidino)-carbonyloxy-camptothecin against human tumor xenografts: lack of cross-resistance *in vivo* in tumors with acquired resistance to the topoisomerase I inhibitor 9-dimethylaminomethyl-10-hydroxycamptothecin. *Cancer Res* **54**: 2823–2829

Jansen WJM, Pinedo HM, Kuiper CM, Lincke C, Bamberger U, Heckel A and Boven E (1994) Biochemical modulation of 'classical' multidrug resistance by BIBW22BS, a potent derivative of dipyridamole. *Ann Oncol* **5**: 733–739

Jansen WJM, Zwart B, Hulscher TM, Giaccone G, Pinedo HM and Boven E (1997*a*) CPT-11 in human colon cancer cell lines and xenografts: characterization of cellular sensitivity determinants. *Int J Cancer* **70**: 335–340

Jansen WJM, Kolfshoten GM, Erkelens CAM, Van Ark-Otte J, Pinedo HM and Boven E (1997*b*) Antitumor activity of CPT-11 in experimental human ovarian cancer and human soft tissue sarcoma. *Int J Cancer* (in press)

Juan CC, Hwang J, Liu AA, Whang-Peng J, Knutsen T, Huebner K, Croce CM, Zhang H, Wang JC and Liu LF (1988) Human DNA topoisomerase I is encoded by a single-copy gene that maps to chromosome region 20q12–13.2. *Proc Natl Acad Sci USA* **85**: 8910–8913

Kaneda N and Yokokura T (1990) Nonlinear pharmcokinetics of CPT-11 in rats. *Cancer Res* **50**: 1721–1725

Kunimoto T, Nitta K, Tanaka T, Uehara N, Baba H, Takeuchi M, Yokokura T, Sawada S, Miyasaka T and Mutai M (1987) Antitumor activity of 7-ethyl-10-[14-(1-piperidino)-1-piperidino]carbonyloxy-camptothecin, a novel water-soluble derivative of camptothecin, against murine tumors. *Cancer Res* **47**: 5944–5947

Lincke CR, Van Der Bliek AM, Schuurhuis GJ, Van Der Velde-Koerts T, Smit JJM and Borst P (1990) Multidrug resistance phenotype of human BRO melanoma cells transfected with a wild-type human *mdr*1 complementary DNA. *Cancer Res* **50**: 1779–1785

Ling V (1992) P-glycoprotein and resistance to anticancer drugs. *Cancer* **69**: 2603–2609

Liu LF and Miller KG (1981) Eukariotic DNA topoisomerases: two forms of type I DNA topoisomerases from HeLa cell nuclei. *Proc Natl Acad Sci USA* **78**: 3487–3491

Lokiec F, Monegier du Sorbier B and Sanderink GJ (1996) Irinotecan (CPT-11) metabolites in human bile and urine. *Clin Cancer Res* **2**: 1943–1949

Mattern MR, Hofmann GA, Polsky RM, Funk LR, McCabe FL and Johnson RK (1993) *In vitro* and *in vivo* effects of clinically important camptothecin analogues on multidrug-resistant cells. *Oncol Res* **5**: 467–474

Niwa K, Misao R, Hanabayashi T, Morishita S, Murase T, Itoh M, Itoh N, Mori H and Tamaya T (1994) Semi-quantitative analysis of DNA topoisomerase-I mRNA level using reverse transcription-polymerase chain reaction in cancer cell lines: its relation to cytotoxicity against camptothecin derivative. *Jpn J Cancer Res* **85**: 869–874

Rogan AM, Hamilton TC, Young RC, Klecker RW and Ozols RF (1984) Reversal of adriamycin resistance by verapamil in human ovarian cancer. *Science* **224**: 994–996

Slichenmyer WJ, Rowinsky EK, Donehower RC and Kaufmann SH (1993) The current status of camptothecin analogues as antitumor agents. *J Natl Cancer Inst* **85**: 271–291

Tsuji K, Kaneda N, Kado K, Yokokura T, Yoshimoto T and Tsuru D (1991) CPT-11 converting enzyme from rat serum: purification and some properties. *J Pharmacobio Dyn* **14**: 341–349

Tsuruo T, Matsuzaki T, Matsushita M, Saito H and Yokokura T (1988) Antitumor effect of CPT-11, a new derivative of camptothecin, against pleiotropic drug-resistant tumors in vitro and in vivo. *Cancer Chemother Pharmacol* **21**: 71–74

Versantvoort CHM, Broxterman HJ, Feller N, Dekker H, Kuiper CM and Lankelma J (1992) Probing daunorubicin accumulation defects in non-P-glycoprotein expressing multidrug-resistant cell lines using digitonin. *Int J Cancer* **50**: 906–911

Wall ME, Wani MC, Cook CE, Palmar KH, McPhail AT and Sim GA (1966) Plant antitumor agents. I. The isolation and structure of camptothecin, a novel alkaloid leukemia and tumor inhibitor from *Camptotheca acuminata*. *J Am Chem Soc* **88**: 3888–3890

Woessner RD, Eng WK, Hofmann GA, Rieman DJ, McCabe FL, Hertzberg RP, Mattern MR, Tan KB and Johnson RK (1992) Camptothecin hyper-resistant P388 cells: drug-dependent reduction in topoisomerase I content. *Oncol Res* **4**: 481–488

Zaman GJR, Versantvoort CHM, Smit JJM, Eijdems EWHM, De Haas M, Smith AJ, Broxterman HJ, Mulder NH, De Vries EGE, Baas F and Borst P (1993) Analysis of the expression of MRP, the gene for a new putative transmembrane drug transporter, in human multidrug resistant lung cancer cell lines. *Cancer Res* **53**: 1747–1750

Zaman GJR, Flens MJ, Van Leusden MR, De Haas M, Mülder HS, Lankelma J, Pinedo HM, Scheper RJ, Baas F, Broxterman HJ and Borst P (1994) The human multidrug resistance-associated protein MRP is a plasma membrane drug-efflux pump. *Proc Natl Acad Sci USA* **91**: 8822–8826

Zamora JM, Pearce HL and Beck WT (1988) Physical–chemical properties shared by compounds that modulate multidrug resistance in human leukemic cells. *Mol Pharmacol* **33**: 454–462

© Cancer Research Campaign 1998